# CONSORT recommendations in abstracts of randomised, controlled trials on migraine and headache

**DOI:** 10.1007/s10194-011-0361-1

**Published:** 2011-06-28

**Authors:** Peer Carsten Tfelt-Hansen

**Affiliations:** Department of Neurology, Danish Headache Center, Glostrup Hospital, University of Copenhagen, 2000 Glostrup, Denmark

**Keywords:** CONSORT statement, Migraine, Treatment, Randomised, Clinical trials

## Abstract

A CONSORT statement on the content of abstracts of randomised, controlled trials (RCTs) was published in 2008. I therefore reviewed the abstracts from 2009 to 2010 published on RCTs in Cephalalgia, Headache and other (non-headache) journals. The following items were reviewed: number of patients, reporting of response either in percentages or absolute values, the use of *p* values, and effect size with its precision. The latter was recommended in the CONSORT statement. A total of 46 abstracts were reviewed and effect size with 95% confidence intervals was only reported in seven abstracts. The influence of the CONSORT statement on reporting in abstracts has so far only had a limited influence on the headache literature.

“For clinical trials, clear, transparent, and sufficiently, detailed abstracts of journal articles and conference abstracts are important because readers often base their, assessment of a trial on such information” Hopewell et al. [1].

## Introduction

As explained in the vignette, the abstract is an important part of the publication of a randomised, controlled trial (RCT). In 2008, the CONSORT group published a statement on reporting RCTs in journal and conference abstracts [1], see Table 1.

**Table 1:** Items to include when reporting of randomised trials in journal or conference abstracts [1]

Item	Description
Title	Identification of the study as randomised
Authors^a^	Contact details for the corresponding author
Trial design	Description of the trial design (e.g. parallel, cluster, non-inferiority)
Methods	
Participants	Eligibility criteria for participants and the settings in which the data were collected
Interventions	Interventions intended for each group
Objective	Specific objective or hypothesis
Outcome	Clearly defined primary outcome for this report
Randomisation	How participants were allocated to interventions
Blinding (masking)	Whether or not participants, care givers and those assessing the outcomes were blinded to group assignment
Results	
Numbers randomised	Number of participants randomised to each group
Recruitment	Trial status
Numbers analysed	Number of participants analysed in each group
Outcome	For the primary outcome, a result for each group and the estimated effect size and its precision
Harms	Important adverse events or side effects
Conclusions	General interpretation of the results
Trial registration	Registration number and name of trial register
Funding	Source of funding

^a^For conference abstracts

I therefore wanted to investigate whether this CONSORT statement has had an impact on the literature on RCTs in migraine and headache treatment. The years 2009 and 2010 were chosen as the appropriate years to evaluate this question. The CONSORT statement for abstract is very demanding (see Table 1) and I therefore chose to review only the most important efficacy items (in italics in Table 1).

## Methods

The three headache journals, Cephalalgia, Headache and Journal of Headache and Pain, were hand-searched twice for RCTs in 2009 and 2010. In addition, PubMed was searched for RCTs in other journals in 2009 and 2010 with the search terms: “migraine”, “treatment” and “clinical trial” as well as “headache”, “treatment” and “clinical trial”. The abstracts were rated for the presence of numbers in each treatment group or total number of patients, percentage response or absolute values for response, *p* values, absolute effect size (percentage responding in active treatment group minus percentage responding in control group) and 95% confidence intervals (95% CI) for absolute effect size (see Tables 2, 3 and 4).

**Table 2:** Presentation in abstracts concerning efficacy in double-blind, randomised, controlled trials (RCTs) in Cephalalgia in 2009 and 2010

References	Numbers in each group (total number of patients)	% response or absolute values (AV)	*p* values	Effect size	95% CI for effect size
2010					
[2]	37/37/38	–	–	–	–
[3]	(1677)	–	+	–	–
[4]	42 CO	–	+	–	–
[5]	343/347	+	+	–	–
[6]	88/42	+	+	–	–
[7]	(117)	+	+	–	–
[8]	30 CO	AV	+	–	–
[9]	347/358	AV	+	–	–
[10]	341/338	AV	+	–	–
[11]	(27)	–	–	–	–
2009					
[12]	–	–	–	–	–
[13]	(859)	+	–	–	–
[14]	(410)	AV	+	–	–
[15]	(95)	AV	+	–	–
[16]	1135/846^a^	+	+	–	–
[17]	58/65	AV	+	+	+
[18]	40 CO	AV	+	–	–

*CO* crossover

^a^Pooled results of 2 RCTs

**Table 3:** Presentation in abstracts concerning efficacy in double-blind, RCTs in Headache in 2009 and 2010

References	Numbers in each group (total number of patients)	% response or absolute values (AV)	*p* values	Effect size	95% CI for effect size
2010					
[19]	177/169	+	+	−	−
[20]	688/696	AV	+	−	−
[21]	99/96	+	+	−	−
[22]	(52)	−	−	−	−
2009					
[23]	19/17	+	+	−	−
[24]	(179)	AV	+	−	−
[25]	153/153	+	+	−	−
[26]	121 CO	+	+	+	+
[27]	(283)	+	+	−	−
[28]	(180)	AV	+	−	−
[29]	(69)	+	+	−	−
[30]	(323)	+	+	−	−
[31]	(60)	+	+	−	−

*CO* crossover

**Table 4:** Presentation in abstracts concerning efficacy in double-blind, RCTs in other (non-headache) journals in 2009 and 2010

References	Numbers in each group (total number of patients)	% response or absolute values (AV)	*p* values	Effect size	95% CI for effect size
2010					
[33]	133 CO	AV	+	−	−
[34]	46 CO	AV	+	+	+
[35]	53/55/55/65	AV	−	+^a^	+^a^
[36]	(196)	AV	−	+	+
[37]	82/82	+	+	+	+
[38]	(66)	AV	−	+	+
[39]	(265)	+	+	−	−
2009					
[40]	117/381/371/365	−	+	−	−
[41]	29/49	AV	+	−	−
[42]	(127)	AV	+	−	−
[43]	31 CO	+	−	−	−
[44]	311/310	+	−	−	−
[45]	172/159	AV	+	+	+
[46]	−	−	+	−	−
[47]	35/35/33	+	−	−	−
[48]	50/50	−	−	−	−

*CO* crossover

^a^Mean and 95% CI for changes from baseline

## Results

In Cephalalgia, 17 abstracts on RCTs (Table 2) [2–18] and in Headache 13 abstracts on RCTs were found (Table 3) [19–31]. In the Journal of Headache and Pain, only one RCT was found (an RCT on deep brain stimulation in 11 patients with chronic cluster headache [31]). In the other (non-headache) journals, I found 16 abstracts of RCTs on headache and migraine [32–47].

The number of patients in each RCT varied from 27 to 1,981 with a median of 180 subjects. Percentage response or absolute values for response were reported in 35 of 46 abstracts (Tables 2, 3, 4) and *p* values were reported in 33 of 43 abstracts (Tables 2, 3, 4). In contrast, effect size and its precision (95% CI) were only reported in the abstract of one RCT in Cephalalgia [16] and Headache [25]. In other (non-headache) journals, effect size with 95% CI was presented in five abstracts [34–37, 44] (Table 4).

## Comments

The number of patients treated in each RCT varied from relatively small crossover trials (minimum, *n* = 27 trials [11] was, however, a parallel-group trial) to very large parallel-group trials (maximum, *n* = 1981). The median was 180 patients, most likely a reasonable number.

In eight papers on RCTs, there was no mention in the abstract of response either in percentages or in absolute values [2–4, 12, 22, 40, 46, 48]. Two of these abstracts were remarkable [3, 40]. One was a very large RCT in which 1,677 patients were treated for >1 attack and 1,263 were treated for all 4 attacks [3]. Based on attack I data, telcagepant 140 and 280 mg were significantly (*p* < 0.001) more effective than placebo for 2-h pain freedom and six other efficacy measures [3]. In the other RCT (*n* = 1,234) with different doses of telcagepant and placebo, only *p* values (*p* < 0.001) were given [39]. These abstracts would not have been made much longer by reporting the responses, e.g. 24 and 25% 2-h pain freedom for telcagepant versus 10 and 11% pain freedom for placebo [3, 39].

*p* values are traditionally used in reporting the results of RCTs and were used in most abstracts. These *p* values can, however, be very small if in a very large RCT there is a small but clinically insignificant difference between two treatments. *p* values can thus sometimes be misleading.

There is generally little reporting of effect size and its precision, which was only presented in seven abstracts [17, 26, 34, 36–38, 45]. Effect size (active minus control) in percentages or absolute value, with 95% confidence intervals (CI), is the clinically relevant measure. It is also useful in “negative” RCTs where 95% CI (and not *p* values) gives the precision of the comparability. Reporting of outcome measures in the abstracts of the 43 papers is thus not optimal when compared with the CONSORT statement for reporting in abstracts [1].

In the latest CONSORT statement from 2010, for efficacy measures with binary outcomes it is recommended that both absolute and relative effect sizes should be presented with an estimate of the precision such as 95% CI [48, 49]. The relative risk (active/placebo) is 1.5 (25%/10%) for pain freedom at 2 h for telcagepant 280 mg and the odds ratio is 3.0 [3]. Relative risk and odds ratio [2] are thus difficult to judge clinically. One should be content with reporting effect size and its precision in abstracts of RCTs on migraine and headache. For example, the effect size for telcagepant 280 mg for pain freedom at 2 h should be reported as 15 with 95% CI: 10–19% [3].

In conclusion, the CONSORT statement from 2008 on reporting RCT in abstracts [1] has only had a minor impact on the headache literature in 2009 and 2010.
